# Parametric optimization of the production of cellulose nanocrystals (CNCs) from South African corncobs via an empirical modelling approach

**DOI:** 10.1038/s41598-022-22865-y

**Published:** 2022-11-04

**Authors:** Olawumi O. Sadare, Nomthandazo Mabunda, Ugochukwu M. Ikegwu, Molly K. Keitemoge, Michael O. Daramola, Kapil Moothi

**Affiliations:** 1 Department of Chemical Engineering, Faculty of Engineering the Built Environment, Doornfontein Campus, University of Johannesburg, PO Box 17011, Johannesburg, 2028 South Africa; 2 Department of Chemical and Biological Engineering, University of Wisconsin-Madison, 1415 Engineering Drive, Madison, WI 53706 USA; 3 Department of Chemical Engineering, Faculty of Engineering, Built Environment and Information Technology, University of Pretoria, Hatfield, Pretoria, 0028 South Africa

**Keywords:** Environmental sciences, Nanoscience and technology

## Abstract

In this study, cellulose nanocrystals (CNCs) were obtained from South African corncobs using an acid hydrolysis process. The delignification of corncobs was carried out by using alkali and bleaching pretreatment. Furthermore, the Box-Behnken Design (BBD) was used as a design of experiment (DOE) for statistical experimentations that will result in logical data to develop a model that explains the effect of variables on the response (CNCs yield). The effects (main and interactive) of the treatment variables (time, temperature, and acid concentration) were investigated via the response methodology approach and the obtained model was used in optimizing the CNCs yield. Surface morphology, surface chemistry, and the crystallinity of the synthesized CNC were checked using scanning electron microscopy (SEM), a Fourier Transform Infra-red spectroscopy (FTIR), and an X-ray diffraction (XRD) analysis, respectively. The SEM image of the raw corncobs revealed a smooth and compact surface morphology. Results also revealed that CNCs have higher crystallinity (79.11%) than South African waste corncobs (57.67%). An optimum yield of 80.53% CNCs was obtained at a temperature of 30.18 °C, 30.13 min reaction time, and 46 wt% sulfuric acid concentration. These optimized conditions have been validated to confirm the precision. Hence, the synthesized CNCs may be suitable as filler in membranes for different applications.

## Introduction

The use of waste biomass in CNCs production could assist in the attainment of zero-waste generation, thereby reducing waste management issues and environmental concerns in the process^1,2^. Inadequate handling, poor collection, and unethical behaviour are major concerns in waste management. Moreover, improper disposal of industrial waste can disturb the natural ecosystems, and destroy or cause harm to species, resulting in the release of harmful pollutants into the environment. About 54.2 million tons of industrial, municipal, and commercial waste are estimated to be produced in South Africa annually. However, only one-tenth of the 54.2 million tons of the generated waste is recycled or repurposed each year, while the remaining fraction ends up in the landfill or burnt^3^.

According to the World Bank, waste generation will reach 3.40 billion metric tons by 2050. Currently, waste reprocessing and composting account for around 13.5 percent of the world's waste^4^. Therefore, recycling waste materials will help in completing the loop in a circular economy. Whenever possible, waste disposal should be eliminated, and where it is inevitable, it must be properly controlled to safeguard human health and protect the environment from being contaminated^5^. Lignocellulosic biomass is an example of agro-waste. Lignocellulosic biomass is made up of cellulose, hemicellulose, and lignin, which constitute the plant cell wall. Lignocellulosic biomass includes corncobs, maize husks, rice husks, stems of cereal plants, nutshells of plant fruits, and many more. Meanwhile, majority of these agricultural or garden wastes are disposed of in landfills or burnt, resulting in environmental pollution^6^.

South Africa is one of the largest producers of corn and about 9 million tons of corncob are produced yearly, generating about 20% of the corn residue^7^. A developing country like South Africa is facing numerous challenges relating to the disposal of waste. The causes of waste disposal challenges include, low investment in infrastructure, lack of support by the government, poor political interest, and wrong attitude of the community concerning the disposal of solid waste etc. Thus, different sources of biomass may be viable materials for cellulose production in the nearest future. In addition, non-woody plants have a competitive advantage, due to their low lignin content, shorter growing period, adequate watering requirements, annual renewability, and high yearly cellulose output. Moreover, there are limitless prospects for non-woody biomass to be harvested, as their use endangers no human activity. Instead, they create a better and more sustainable environment.^6^.

Interestingly, CNCs are derived from cellulose isolated from agricultural, industrial wastes, plant-based wastes, etc.^8^. CNCs are widely used as advanced materials and matrices in the papermaking industry. For example, CNCs are used as supporting fillers for polymers, shape memory polymers, healable polymeric materials, food industry, drug carriers in the pharmaceutical industry, supporting matrix for catalysts, and nanomedicine^9^. Owing to their novel abilities, CNCs are extensively utilized in applications that require surface modification of hydroxyl groups. On the other hand, the unique qualities of CNCs, such as, the large surface area, extremely reactive hydroxyl groups, isolation from natural sources, sustainability, renewability, easy production approaches, and their utilization as complex matrixes and materials have drawn the attention of scientists. Furthermore, as a result of their exceptional chemical and mechanical properties, nanometric sizes, and high aspect ratio, CNCs have several prospective applications in a wide range of disciplines, such as medicine, electronics, and material science.

Generally, acid hydrolysis is a well-known technique for the isolation of CNCs, but the yield is usually limited to a lab-scale use. Therefore, process optimization of the parameters is essential for higher yield of CNCs and improved crystallinity index^10^. Application of DOE is necessary to study the parametric effect during the synthesis of CNCs, to understand both the main and interactive effect of the synthesis variables. DOE is expressed as a logical and effective technique that allows researchers and engineers to study the interaction between several input variables^11^. As a matter of fact, many researchers have utilized Central Composite Designs (CCD) as an example of DOE to design experiments, to investigate the effect of variables during the recovery of CNCs from waste. For instance, Wijaya et al.^12^ studied parametric optimization of the production of CNC from Bamboo shoots via response surface methodology. It was reported that about 50.61% CNCs yield yield was obtained within the identified constraints (synthesis variables). In addition, Thakur et al.^13^ investigated the optimization of the synthesis of CNC from rice straw via sulfuric acid hydrolysis, using a Response Surface Methodology (RSM) approach. The authors used CCD for the design of the experiment. Consequently, an improved yield of CNCs (90.28%) was obtained with optimum synthesis conditions being 30 °C temperature, 75 wt% acid concentration and 5 h reaction time. Similarly, García-Garcí et al.^14^ investigated the parametric optimization of hydrolysis during the production of CNCs from pine cones. The authors reported 15% as the maximum yield and the physicochemical properties of the produced CNCs at the optimum hydrolysis conditions were reported as a function of the thermal stability, crystallinity, and aspect ratio (diameter/length). Furthermore, Demewoz^15^ studied parametric optimization of the production of CNCs from corncobs using a response surface methodology. The authors used CCD for the design and synthesis variables considered were acid concentration, hydrolysis temperature, and hydrolysis time. A CNCs yield of 40.94% was reported by the authors at 61.66 wt% acid concentrations, synthesis temperature 45 °C, and at a hydrolysis time of 59.92 min. In the same vein, Demewoz^15^ utilized a CCD to study the parametric optimization of the isolation of CNCs from waste corncob and the authors used CCD with axial points outside the cube as the DOE. On the contrary, Banza and Rutto^16^ recently used the BBD as the design of the experiment to study the parametric optimization of the acid hydrolysis of waste millet husk into CNCs. Within the experimental constraints, a yield of 93.12% was reported by the authors at a sulfuric acid concentration of 65 wt%, a hydrolysis temperature of 50 °C, and a hydrolysis time of 10 min.

In spite of this report, parametric optimization of the production of CNCs from waste corncob using BBD as the DOE has not been extensively investigated and reported in the open literature. Whenever CCD is used as the DOE, the points are usually not of major concern, because they are outside of safe operational limits. However, in BBD, axial points are not considered in the design, but all design points are within a safe working zone. Furthermore, BBD makes sure that no factor is concurrently fixed to its optimum value. BBD usually contains fewer design points with the same number of elements, than CCD, indicating that the operation could be cost-effective. The advantage of BBD over CCD is in addressing the problem of experimental boundaries position. Though CCD is employed frequently, BBD is a good design because the quadratic model can be well fitted. Furthermore, the choice of the design technique was as a result of its ability to allow multiple responses, and to avoid conducting experiments under extreme conditions. As far as it can be ascertained, studies using the BBD as a DOE in parametric optimization during the production of CNCs from waste South African corn cobs are limited in open literature. Moreover, only one or two experimental conditions are varied in the literature using the CCD technique. However, in this recent study, interactive effects of three experimental variables (hydrolysis time, sulfuric acid concentration, the hydrolysis temperature) on the yield of CNCs were investigated. Thereby, allowing numerous applications of CNCs obtained from waste at an industrial scale, at a minimized cost.

Against this background, BBD was used in this study as a DOE for statistical experimentations that will result in logical data to develop a model that explains the effect of variables on the response (CNCs yield). Three operating conditions, namely, the hydrolysis time, the hydrolysis temperature, and the concentration of the sulfuric acid, were considered as the hydrolysis variables in this study, following the reports of Wijaya et al.^12^ and Garcı´a-Garcı et al.^14^ to understand the hydrolysis of South African corncobs into CNCs for possible application on an industrial scale.

## Materials and methods

Some quantity of corncob of South African origin was purchased from City Deep market (Coordinate: − 26.229119, 28.082659) in Johannesburg, South Africa. Sulfuric acid (H_2_SO_4_, 98%), Sodium hydroxide (NaOH, 98%), glacial acetic acid, and aqueous sodium chlorite were procured from Protea Chemical (Pty) located in South Africa. Deionized (DI) water was produced in-house and was used in all the experiments. All chemicals were used without further purification. All methods were performed in accordance with the relevant guidelines and regulations.

### Corncobs pre-treatment and delignification of cellulose

The corncob samples were first crushed into small sizes and then pulverized into a fine powder using a hammer mill (Trojan TGS210E 4HP 220 V, South Africa). About 40 g pulverized corncob was mixed with a 4% (w/w) sodium hydroxide at a liquid-to-solid mass ratio of 20:1 aqueous solution at 80 °C, under mechanical stirring at 500 rpm for 2 h^17^. This was performed for the removal of the hemicellulose and the lignin from the corncob and for cellulose purification^13^. The alkaline suspension obtained was later vacuum-filtered and washed severally with deionized water to ensure complete removal of the alkali. The alkali-treatment step was repeated four times^18^, and the product was dried in an air-circulating oven at 50 °C for 24 h and allowed to go through the bleaching process for color whitening.

To bleach the alkali-treated corncobs, acetate buffer solution (v: v) was prepared by diluting 27 g NaOH and 75 mL glacial acetic acid (CH_3_COOH), in 1L of deionized water and aqueous sodium chlorite (1.7 wt%). The ratio of the alkali-treated powder to liquid was 1:20 (g/mL) as described in another study^19^. To remove the lignin and any other organic residues, the solution was treated at a temperature of 70 °C for 4 h in a water bath, under constant agitation at a speed of 600 rpm. The suspension was then vacuum-filtered, and the solid was washed repeatedly with deionized water to remove the yellow colour and attain a neutral pH^19^. Afterward, the wet residues were dried in an air-circulating oven, at 50 °C for 12 h. Using the same conditions, this step was repeated three consecutive times, and the resulting material after the purification was corncob cellulose. Finally, the dried corncob cellulose was kept in an air-tight bag.

### Preparation of CNCs

**Figure 1:**
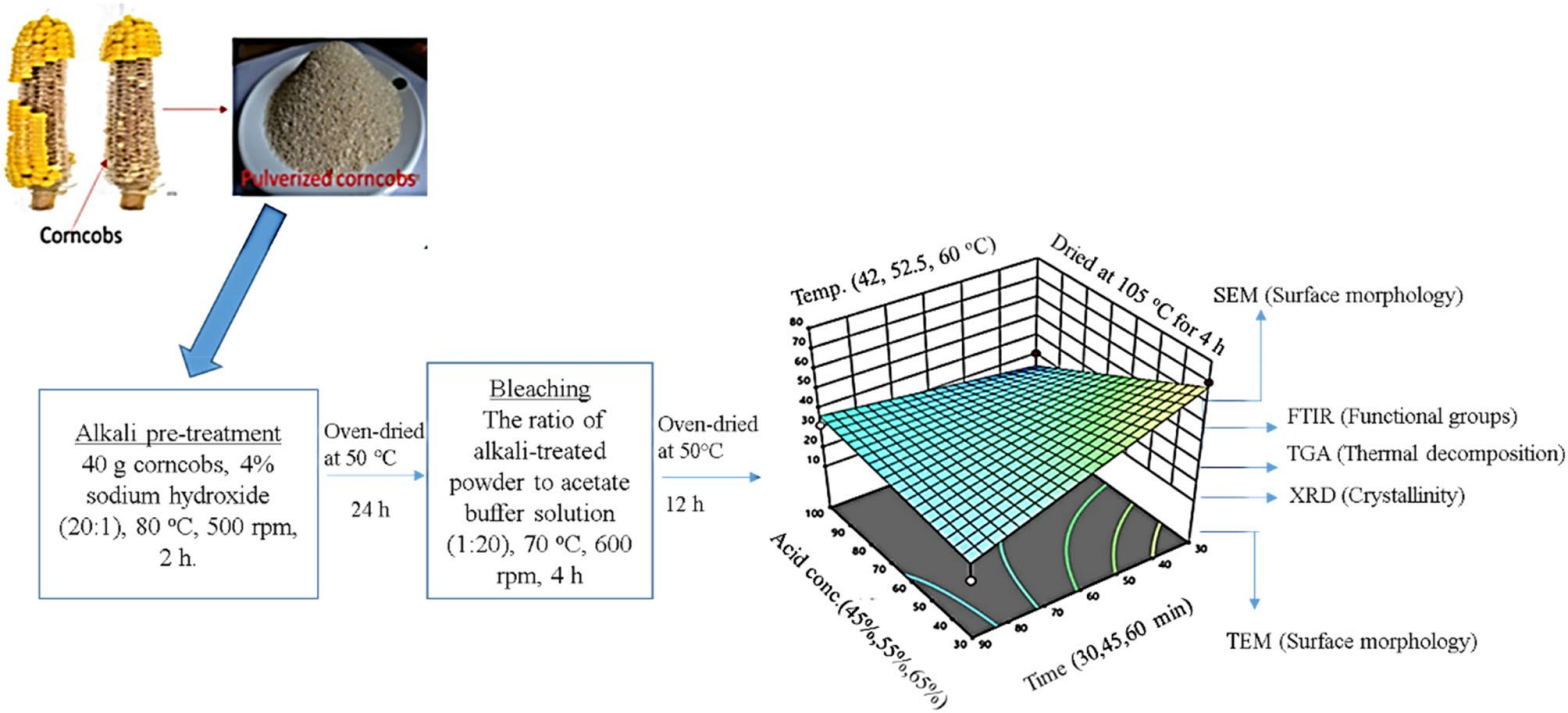
Schematic diagram of the preparation of CNCs from waste South African corncobs.

A ratio of 1:15 (g/mL) corncob-based cellulose to the acid solution was used to prepare the CNCs^9^, and the three most significant factors were carefully chosen. The corncob cellulose was treated with sulfuric acid, and the experiment was carried out using different hydrolysis times, reaction temperatures, and acid concentrations. The mixture was stirred continuously until the end of the hydrolysis time^20^. The experimental variables used in this study are; acid concentration percentage (45%, 55%, 65%), reaction temperature (45, 52. 5, and 60 °C), and reaction time (30, 45, and 60 min) with constant cellulose to acid (1:15 g/mL) solution under vigorous and constant agitation in the water bath. To stop the hydrolysis reaction, 1500 mL of distilled water was added to the suspension, and then the diluted suspensions were allowed to cool down to room temperature. The resulting cellulose nanocrystal was thoroughly rinsed with deionized water and re-centrifuged three times until the suspension reached a pH of 7, and dried at 105 °C for 4 h. The synthesized CNC was kept in an air-tight bag to avoid moisture absorption and kept in a safe environment for characterization. The schematic diagram of the synthesis of CNCs from waste South African corncobs is presented in Fig. 1.

### Parametric effect and optimization of acid hydrolysis of waste corncob-derived cellulose

#### RSM approach using BBD design

RSM is an approach that employs a validated empirical model to study the parametric effect of variables on a system. In this study, STATISTICA software (Stat-Soft, USA, version 10) was used to design the experiments^21^. The three variables, the initial concentration of sulphuric acid, temperature, and reaction time, were considered for the DOE. Each variable was varied at two levels: A: Temperature (30–100 °C), B: Time (30–90 min), and C: Sulphuric acid concentration (45–65 wt%). The response was the yield of CNCs (only considering the quantity of CNC produced relative to the cellulose’s initial weight. The independent variables and levels for BBD are shown in Table 1. Under normal conditions, experiments were performed in triplicate for the accuracy of data. The design of the experiment led to a total of 17 experiments. Data generated from the experiments were used to develop a regression model to describe the observation. One-way analysis of variance (ANOVA) was used to evaluate the validity of the developed model. Statistical quantities such as R-squared, adjusted R-squared, predicted R-squared, and the lack of fit was used to substantiate the aptness and significance of the developed regression model. Finally, the validated model was employed in studying the parametric effect via the RSM approach. In addition, the model was used to study the parametric optimization of acid hydrolysis.

**Table 1:** Independent variables and levels for BBD.

Independent variables	Unit	Actual levels of coded factors		
		− 1	0	1
Temp	°C	30	65	100
Time	Minute	30	60	90
Conc	wt%	45	55	65

### Physio-chemical characterization of raw corncobs and isolated cellulose nanocrystal obtained from waste corncob

The surface morphology of the raw corncob sample and the isolated cellulose nanocrystal from the raw corncob after hydrolysis was checked using a Field Emission Scanning Electron Microscope (TESCAN VEGA, USA). Prior to observation, all CNCs samples were coated with gold, to prevent charge up. Transmission electron microscopy (FEI Titan3 80-300), operated at 300 kV was used to check the surface morphology of the prepared CNCs. The prepared CNCs were placed on carbon-coated copper grids and stained with uranyl acetate. The images of the air-dried CNCs samples were taken by the TEM to get information about the morphology and the particle size of individual particles (It should be noted that agglomerate particles were excluded).

FTIR spectroscopy was used to check the attachment of certain functional groups and much more information on the fiber structure. The FTIR spectra were obtained using a NICOLET iS10 FT-IR Spectrometer in transmittance mode. A 5 mg of CNC powder samples were placed in a KBr matrix and hard-pressed into a pellet. The CNCs samples were examined with a 2 cm^−1^ resolution in a spectral range between 400 cm^−1^ and 4000 cm^−1^ and the resulting spectra were averaged across 32 scans.

X-ray diffraction (Bruker, Germany) was used to study the crystalline structure of corncob and the CNCs. At room temperature, the operating condition was fixed at 40 kV voltage and current of 30 mA^22^, using a sampling pitch of 0.0100 (deg) within a 2θ ranging from 5° to 60^o^ and a scan speed of 1° min^−1^ at a constant scan mode. The crystallinity index (CrI) analysis showed that as the crystallinity of the sample increased, the amorphous region decreased^23^. The crystallinity index (CrI) represents the ratio of the crystalline peak to the amorphous region in the diffractogram based on a monoclinic structure of cellulose and is calculated using Eq. (1)^20^.

1 $$CrI=\frac{{I}_{200}-{I}_{am}}{{I}_{200}}$$

where I_200_ is the peak intensity at the plane (200) and I_am_ is the intensity peak of the amorphous region, at 2θ, is approximately 18°.

Scherrer’s equation was used to determine the size of the CNCs using Eq. (2);

2 $$\mathrm{Crystal size }\left(\mathrm{L}\right)=\frac{\mathrm{k\lambda }}{\mathrm{\beta\, cos \, \theta }}$$

where λ = 0.1540 nm, k is the correction factor of 0.91, θ = diffraction angle in radians and β = full width at half maximum (FWHM)^24^

The thermal degradation properties of the CNCs at different operating variables were analyzed by TA instrument SDT Q600 simultaneous DSC/TGA analyser (TA instrument, New Castle, DE, USA) in the temperature range of 20–700 °C in the same heating rate of 25 °C /min under the nitrogen atmosphere 40 mL/min flowrate.

The gravimetric analysis technique was employed to calculate the CNCs yield. At the end of the experiment, CNCs were dried at 100 °C for 4 h, and the weight of each experimental run was measured. M_2_ is the dry weight of the final dried CNCs sample, while M_1_ is the dry weight of the initial dried raw corncob. The final yield was calculated to make sure the result is accurate and to reduce errors as the average of three parallel experimental runs. The yield of the CNCs sample was obtained using Eq. (3) as follows:

3 $$Yield \left(\%\right)=\frac{{M}_{2}}{{M}_{1}}\times 100\%$$

## Results

### Characterization of synthesized CNCs

#### Fourier transform infrared spectrometry (FTIR)

**Figure 2:**
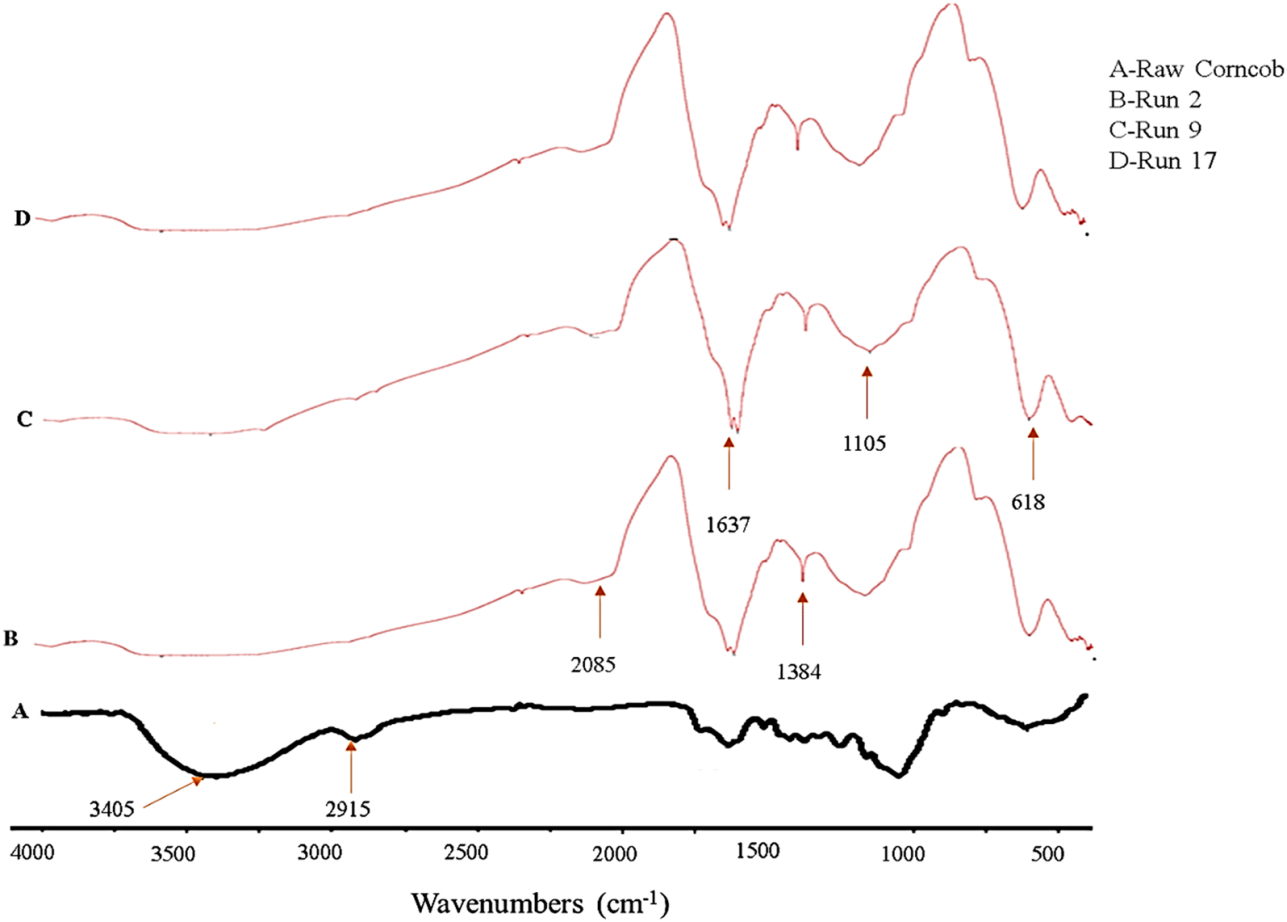
FTIR spectra of raw corncobs and isolated CNCs.

The surface functional groups of the raw corncobs and the prepared CNC samples for experimental runs 2, 9, and 17 are depicted in Fig. 2 (See Table 2 for reaction parameters). The broad peaks at 3405 cm^−1^ were seen in the raw corncob samples, which is related to the hydrogen bond O–H stretching vibration, indicative of the fibers' hydrophilic tendencies^25^. The peak at wavenumber 2915 cm^−1^ of the raw corncobs’ spectrum may be attributed to the C–H stretching for aromatics and alkanes. It could be observed that all the samples for the experimental runs 2, 9, and 17 revealed similar major bands at specific wavenumbers that strongly correlated with the cellulose structure. These observations are in line with previous literature^26^. The absorbance peak for the C–H stretching group was discovered to appear between 3000 cm^−1^ and 2840 cm^−1^. The band at 1085 cm^−1^ signifies the C–O bending of a cellulose. The spectra of all the CNCs samples showed bending variations of C-O groups in polysaccharide rings and symmetrical bending of CH_2,_ at a characteristic absorption band of 1105 cm^−1^. The band at 1637 cm^−1^ corresponds to the OH bending of adsorbed water. On the other hand, the occurrence of sulfate groups in the synthesized CNCs from corncobs via sulfuric acid hydrolysis could be observed at a wavenumber of 1384 cm^−1^. Furthermore, the absence of bands in the spectra of all cellulose nanocrystal samples compared to the raw corncobs at 2915 cm^−1^ and 3405 cm^−1^ could be attributed to the purifying procedure on the corncobs before acid hydrolysis^27^.

**Table 2:** Experimental design based on BBD with the response (Yield %).

Std	Run	A: Temp (°C)	B: Time (minute)	C: Conc. (wt%)	Response 1 Yield %
9	1	65	30	45	50
4	2	100	90	55	32
1	3	30	30	55	70
17	4	65	60	55	36
16	5	65	60	55	36
6	6	100	60	45	46
15	7	65	60	55	36
7	8	30	60	65	31
8	9	100	60	65	23
13	10	65	60	55	36
5	11	30	60	45	80
12	12	65	90	65	25
10	13	65	90	45	48
11	14	65	30	65	28
3	15	30	90	55	18
14	16	65	60	55	36
2	17	100	30	55	31

#### Surface morphology of raw corncobs and synthesized CNCs

**Figure 3:**
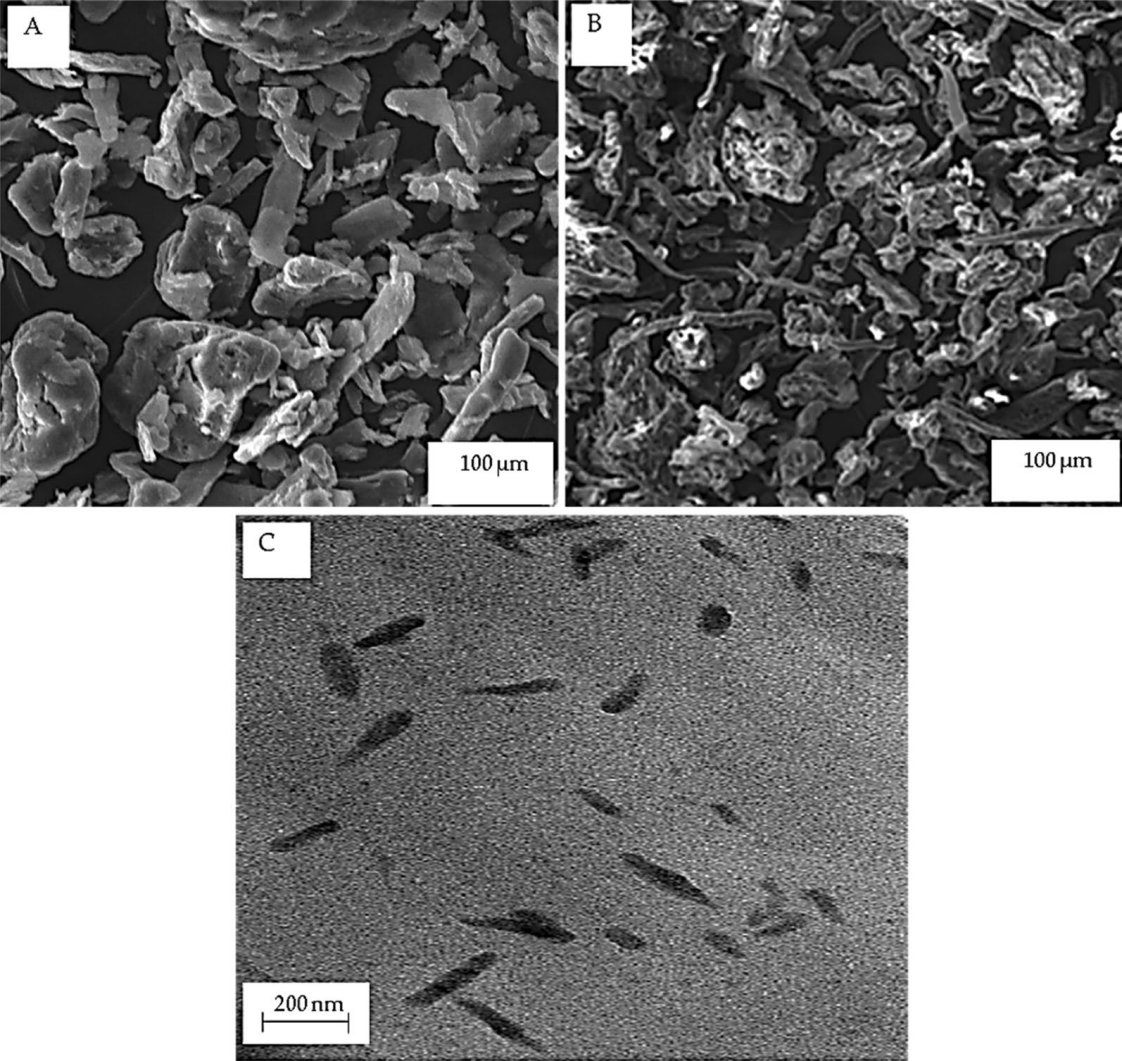
(**A**) SEM image of alkaline-pretreated corncobs (**B**) SEM image of corncobs cellulose (**C**) TEM image of prepared CNCs.

Figure 3A,B depict the surface morphology of alkaline-treated corncobs at 20 µm and of corncobs cellulose at 100 µm, respectively at 1 kx and × 300 magnification. The image in Fig. 3A showed a smooth and irregular structure. The alkaline treatment process is able to disintegrate the hemicellulose, thus the alkaline-treated corncob became water-soluble. Figure 3B depicts the SEM image of the corncob cellulose. It could be observed that the fiber appeared rougher compared to the alkali-treated corncob and it is a micro-sized fiber that displayed an irregular shape. This is an indication that the hemicellulose and the lignin have been effectively removed due to the chemical treatments. In addition, the process of purifying and bleaching seemed not to decompose the cellulose structure. The disintegration of the fiber bundles into individual cells could be observed due to the elimination of lignin during the bleaching process, thus, making the isolation of CNCs from corncob more likely. A similar result was reported^15,28^. TEM image of the CNCs in Fig. 3C showed spherical-shaped nanoparticles^29^ with an average diameter and length of 40 ± 1.11 nm and 245 ± 0.91 nm, respectively. The CNCs were found to conform to nanoscale dimensions (1–100 nm in diameter) with respect to the particle size measurements^30,31^. This spherical shape morphology could be attributed to stacking nanocrystals as a result of the Vander Waals forces.

#### The XRD pattern of the raw corncobs and the synthesized CNC

**Figure 4:**
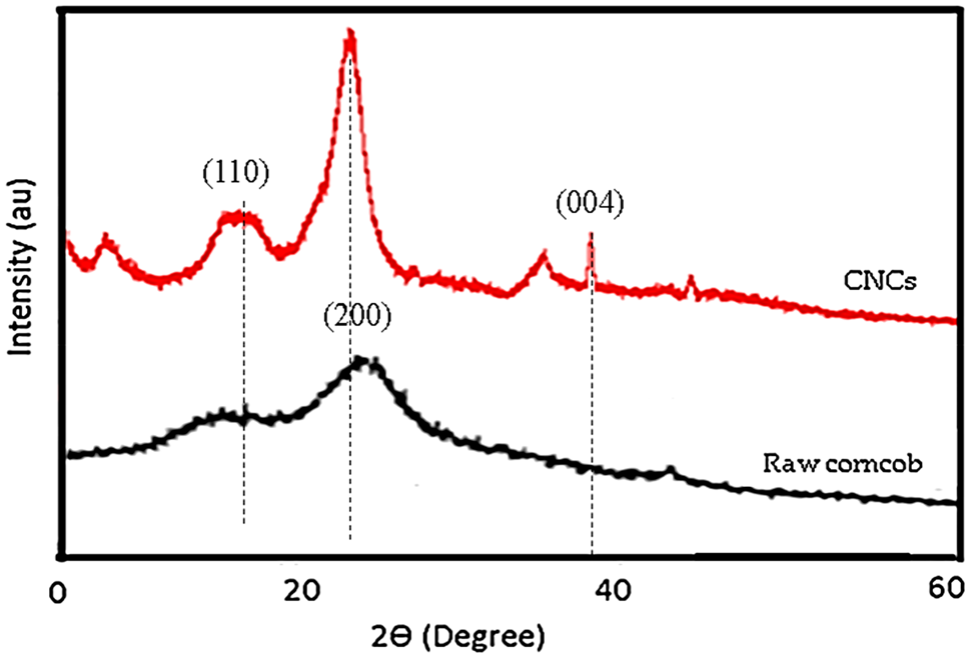
X-ray diffractogram of raw corncob and synthesized CNCs.

Figure 4 illustrates the X-ray diffraction patterns of the raw corncob and the acid-hydrolyzed CNCs to determine their crystalline indexes. As it can be observed in Fig. 4, the CNCs exhibited a sharp and large peak. The spectra for the prepared CNCs contained peaks located at 2θ ~ 16.8°, 2θ ~ 22.7°, corresponding to peaks arising due to reflections from, (110), (200), respectively and 2θ ~ 35.8°, and 2θ ~ 37.9°, corresponding to peaks arising as a result of the reflections from (004) planes of CNCs. This may be attributed to the amorphous parts of the raw corncob that were removed by the pretreatment and the acid hydrolysis. A similar observation was reported by Das et al.^32^. The raw corncob and the synthesized CNCs show peaks at around 2θ = 22°. The unexpected peaks observed at 2θ ~ 5° and 2θ  ~ 45° could be as a result of impurities present in the CNCs samples. The crystallinity indexes (CrI) calculated using Eq. (1) were 57.67% and 79.11% for raw corncob and CNCs, respectively. The higher CrI value obtained for CNCs than raw corncob can be attributed to the degradation and elimination of amorphous non-cellulosic compounds as a result of the acid hydrolysis, alkali, and bleaching treatments during the pretreatment process. The paracrystalline fraction of cellulose is located at the surface of crystallites as thin monomolecular layers. Indeed, structurally the paracrystalline state is closer to an amorphous state but has certain random collinearity between chains. However, the main part that is removed by acid hydrolysis from cellulose is the amorphous region. Therefore, the increased CrI (%) value of CNCs compared to raw corncob could be attributed to the partial removal of the paracrystalline domains during acid hydrolysis. It can be seen that the narrow and sharper peaks for the CNCs, as a result of the acid treatment, contribute to the higher crystallinity of the nanostructures compared to the raw corncobs. This result is similar to what was obtained in the literature^12^. Around 2.41 nm crystallite size was obtained for the CNCs using Eq. (2).

#### Thermogravimetric analysis of corncobs

**Figure 5:**
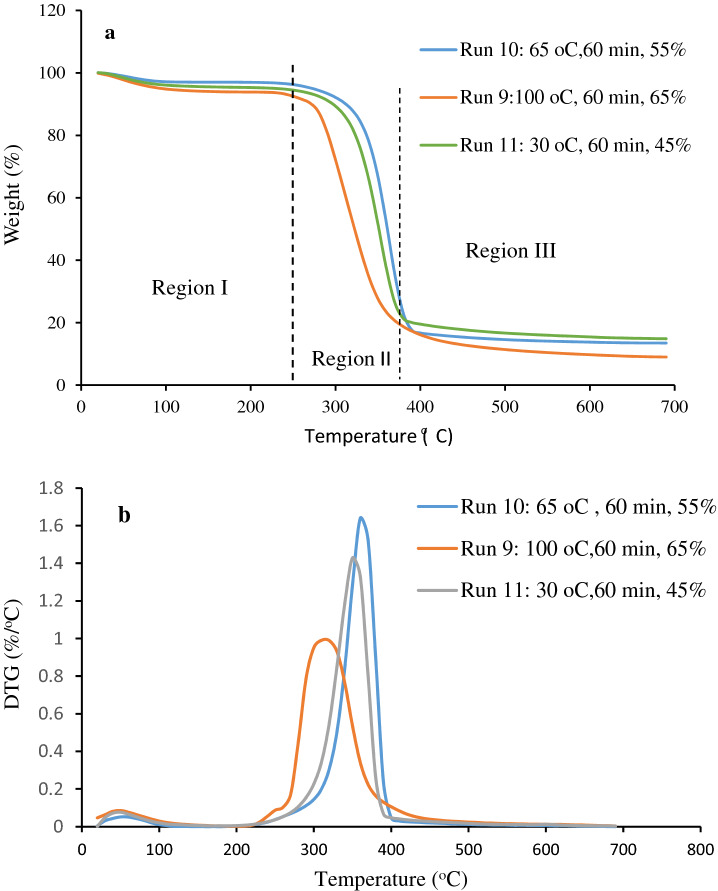
(**a**) TGA (**b**) derivative thermogravimetric (DTG) curves of CNCs at different operating variables.

Figure 5a,b depicts the TGA and Derivative thermogravimetric curves of CNCs samples at different operating variables. Figure 5a indicated that all samples showed the same behavior with three regions of thermal degradation. A slight mass loss was observed at Region I, within the temperature range of 20–240 °C. This is mainly as a result of vaporization of moisture absorbed by all CNCs samples^33^. Furthermore, all samples exhibited the main stage of thermal degradation at a temperature range of 240–380 °C (Region II). This region indicated the maximum thermal degradation among all samples and CNCs underwent stronger depolymerisation. The loss of weight could be due to the thermal degradation of the cellulose, hemicellulose and the lignin components^14^. A further breakdown of thermal degradation intermediates was noticed at Region III around temperature range of 380–700 °C^34^.

From Fig. 5b, it could be observed that the initial temperatures at the main degradation of hydrolyzed CNCs for Run 9, 10 and 11, with different temperatures of 100, 65 and 30 °C were 310, 360, and 350 °C, respectively. This indicated that thermal stability was decreased when the hydrolysis temperature increased. This could be attributed to more crystalline regions of the cellulose that was dissolved during hydrolysis at increased temperature (≥ 100 °C), causing reduced crystallinity and then resulting in reduced thermal resistance. A similar observation was reported by Kusmono et al.^34^. The onset degradation temperature of CNCs made at various acid concentrations of 45, 55 and 65% was 360, 370, and 330 °C (Run 11, 10 and 9), respectively. A similar effect was also exhibited by the acid concentration where the initial temperature at the main degradation of CNCs was also reduced from 370 °C to 330 °C with an increase in acid concentration from 55 to 65%. The lower thermal resistance of CNCs for Run 9 was related to the presence of sulfate groups on the surface of the CNCs that speed up the rate of degradation^35^. This could also be attributed to the presence of more negatively charged sulfate groups on the CNCs surface, due to hydrolysis at increased acid concentration of 65%, resulting in decreased thermal resistance. Kusmono et al.^34^ and Haafiz et al.^36^ also established that the existence of sulfate groups on the surface of CNCs reduced the thermal resistance as a result of dehydration reaction.

### Box–Behnken design

Using the experimental data generated from DOE obtained via BBD, an empirical model that describes the effect of the process variables on the production of CNCs from waste corncob was developed. Among other types of design, BBD offers lower laboratory labor and cost as it provides the smallest number of experimental runs and significantly provides a reliable model design^37^. Subsequently, Response Surface Methodology (RSM) was used to understand both the main effect and interactive effect of the hydrolysis variables on the yield of CNCs. Table 2 shows the experimental results.

### Model development

Equation 4 is the empirical model obtained using the experimental data in Table 2. The model relates the yield of nanocellulose yield to the hydrolysis variables.

The General form of an empirical model is shown in Eq. (4).

4 $$Y{\alpha }_{1}= {\alpha }_{2}A+{\alpha }_{3}B+{\alpha }_{4}C+{\alpha }_{5}AB+{\alpha }_{6}AC+{\alpha }_{7}BC+{\alpha }_{8}{A}^{2}+{\alpha }_{9}{B}^{2}+{\alpha }_{10}{C}^{2}$$

Y is the response (Yield %); A, B, C are the coded variables, and α_i_ are the regression coefficients. Table 3 presents the Analysis of Variance (ANOVA) for the developed model.

**Table 3:** Analysis of variance (ANOVA) for the developed model for cellulose nanocrystal yield model regression.

Source	Sum of squares	d_f_	Mean square	F-value	p-value	
Model	3535.75	6	589.29	9.93	0.0010	significant
A-Temp	561.12	1	561.12	9.46	0.0117	
B- Time	392.00	1	392.00	6.61	0.0279	
C- Conc	1711.12	1	1711.12	28.85	0.0003	
AB	702.25	1	702.25	11.84	0.0063	
AC	169.00	1	169.00	2.85	0.1223	
BC	0.25	1	0.25	0.0042	0.9495	
Residual	593.19	10	59.32			
Lack of fit	593.19	6	98.87			
Pure Error	0.00	4	0.00			
Cor Total	4128.94	16				

As observed in Table 3, the model is significant such that the F-value is greater than the p-value and the p-value is lower than 0.0500, indicating its significance within the 95% confidence limit. Nevertheless, considering each variable, some variables are insignificant to the model, i.e., the p-values is greater than 0.1000. Therefore, the hydrolysis temperature, hydrolysis time, acid concentration, and interaction between temperature and time only (A, B, C, and AB terms) are significant. The statistical significance of these variables shows that the alterations in the independent variables (temperature, reaction time, acid concentration) and the interaction between temperature and reaction time significantly affected the CNCs yield. However, the degree and direction to which each significant variable affect CNCs yield is seen in the coded equations (Eq. 5). The Positive sign in front of each term of the coded Equation denotes a creative effect. Conversely, the negative sign signifies a counter effect. Likewise, a term with a higher coefficient shows a higher magnitude of influence on the yield than a term with a smaller coefficient. Hence, from Eq. (5), it is seen that temperature and acid concentration influenced the CNCs yield more significantly than time. Although, all the three independent terms negatively affected the CNCs yield, Table 3 shows that the yield of CNCs increased at the onset of acid hydrolysis as the concentration of acid increased. This observation could be attributed to the increased infiltration of acid into the amorphous part of cellulose which improved the conversion of cellulose to CNCs. However, the yield was the highest when the acid concentration was 55 wt% and then reduced with an increased acid concentration of up to 65 wt% as a result of the excessive degradation of cellulose to unwanted products. Increasing the temperature above 30 °C decreased the yield values, as attributable to further degradation of the cellulose at a higher temperature above 30 °C. It could be seen that at the highest hydrolysis time (90 min), the lowest yield of CNCs was obtained. This observation may be attributed to the extra hydrolysis time allowed for the acid hydrolysis of the cellulose.

The optimum yield value was achieved when the hydrolysis time was at its minimum value of 30 min. It could also be observed that as the hydrolysis time increased, the cellulose seemed to degrade further, thereby, decreasing the yield of CNCs. The acid concentration had the greatest effect on the acid hydrolysis yield of CNCs in comparison to the reaction time and temperature, as shown in Eq. (5). This observation corroborates the result reported by Wijaya et al.^12^. Equations (5) and (6) are the models with coded values and the actual values, respectively. While Eq. (5) gives the magnitude and direction of each variable impact on the CNCs yield, Eq. (6) was drawn to scale due to the different units to predict these responses. The R^2^ value, the adjusted R^2^, and the predicted R^2^ were 0.8563, 0.7701, and 0.3039. The R^2^ indicates the extent to which the variables are in correlation with the yield. The low R-squared values obtained for the R^2^, adjusted R^2^, and the predicted R^2^ show that even noisy, high-variability data can have a significant trend. The trend shows that the predictor variable still offers information about the CNCs yield (response) even though data points fall further from the regression line. Conversely, the R^2^ increased, as the number of terms increased. Hence, the adjusted R^2^ provides a better explanation of this correlation.

5 $$Y=38.94-8.37A-7.00B-14.63C+13.25AB+6.50AC-0.25BC$$

6 $$Y=261.79-2.02A-1.01B-2.62C+0.01AB+0.02AC-0.0008BC$$

The AC and BC terms are eliminated, since all the quadratic terms are insignificant, as observed in Table 3 (p-value > 0.1000). Therefore, Eqs. (7) and (8) represent the new coded and actual equations, respectively. Although, the interaction between temperature and reaction time and the interaction between temperature and the acid concentration (AC and BC) are insignificant, these terms are retained, sustaining the order in the model equations. These shall be extensively discussed in the subsequent sections. The new one-way ANOVA is presented in Table 4.

**Table 4:** Analysis of variance (ANOVA) of the new model.

Source	Sum of squares	df	Mean square	F-value	p-value	
Model	3366.50	6	687.54	13.2500	0.0002	Significant
A-Temp	561.12	1	561.12	8.8300	0.0117	
B- Time	392.00	1	392.00	6.1700	0.0288	
C- Conc	1711.12	1	1711.12	26.9300	0.0002	
AB	702.25	1	702.25	11.0500	0.0061	
Residual	762.44	12	63.54			
Lack of fit	762.44	8	95.31			
Pure Error	0.00	4	0.00			
Cor Total	4128.94	16				

7 $$Y=37.94-8.37A-7.00B-14.63C+13.25AB$$

8 $$Y=198.15-1.00A-1.05B-1.46C+0.01AB$$

The new R-squared, adjusted R-squared, and predicted R-squared values are 0.8153, 0.7538, and 0.5085, respectively. The R-squared values were considerably improved by removing the insignificant terms from the model. However, the predicted R-squared values were still relatively low. This observation suggests that this model can be considerably improved and robust by increasing the number of experimental runs.

Having highlighted that the coded equations are employed to recognize the relative effect of the factors by comparing the coefficients, while the actual equations predict the actual yield values at varying process conditions. It can be observed that temperature above 30 °C had a negative impact on the yield. This is contrary to the report of Wijaya et al.^12^, that temperatre had no effect on the yield of CNCs. Chen et al.^38^ also suggested that reducing the concentration of sulfuric acid and increasing the reaction time could greatly enhance CNCs yield. However, increasing the acid concentration from 55 wt% to 65 wt% has an adverse effect on CNCs yield and increased the reaction time from 60 to 90 min. The same observation was reported by Matebie et al.^39^.

**Figure 6:**
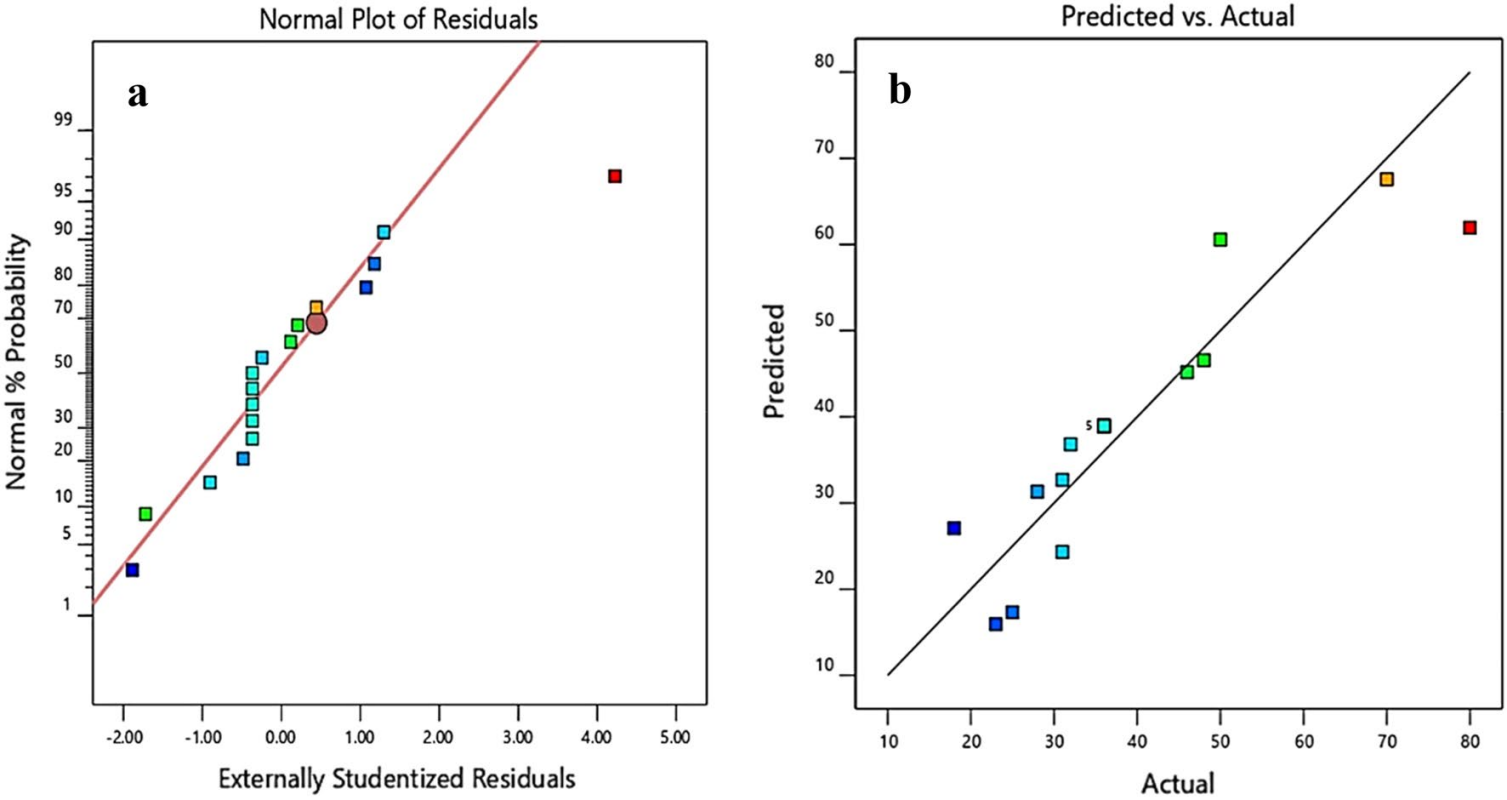
(**a**) Normal plot of residuals (**b**) Actual vs. predicted for CNC yield.

Figures 6a,b depict the normality curve and a plot of the predicted vs. actual responses for CNCs yield, respectively. To confirm the correctness of the model developed, another measure was added by the plot of the actual vs. predicted response as depicted in Fig. 6b, using the actual final model. As depicted in Fig. 6, the data points on the plots can be seen to be evenly distributed, and well sitted within the model line, signifying that low data deviation, as well as normal distribution of errors. Outliers were discovered, with only a small number of projections varied significantly from the actual values, in line with the outliers. Nevertheless, the closeness of the outliers to the straight line indicates that the model assumptions are correct, even though with insignificant errors^13^.

### Interactional effect of process conditions using response surface methodology

**Figure 7:**
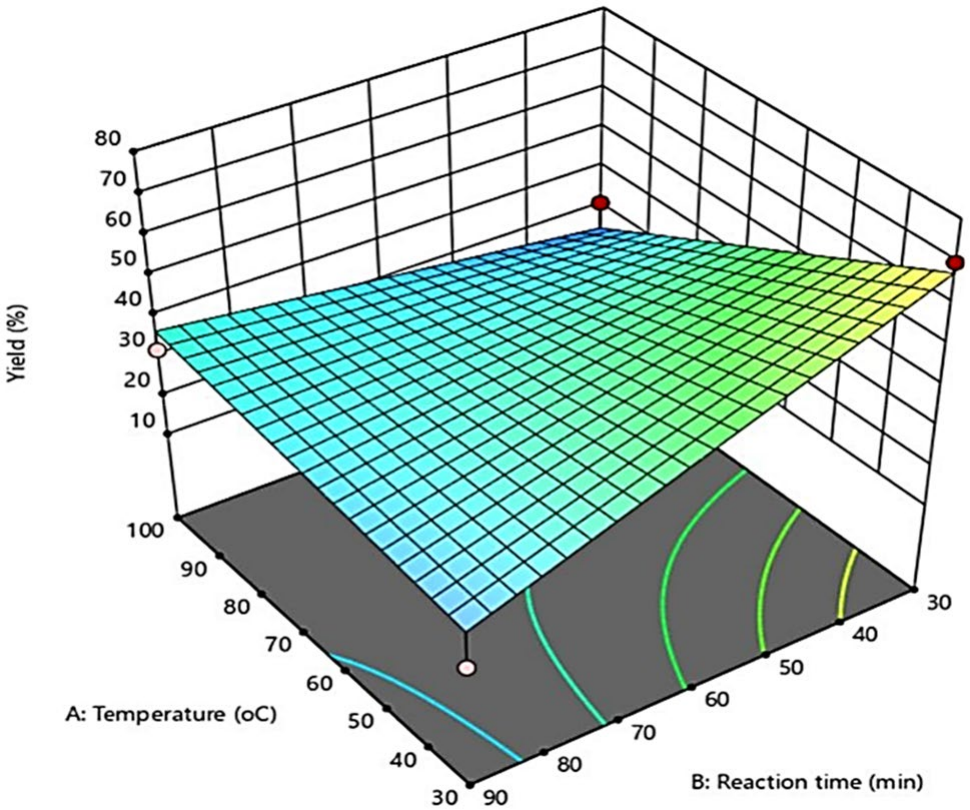
Interaction effect between hydrolysis temperature and hydrolysis time.

**Figure 8:**
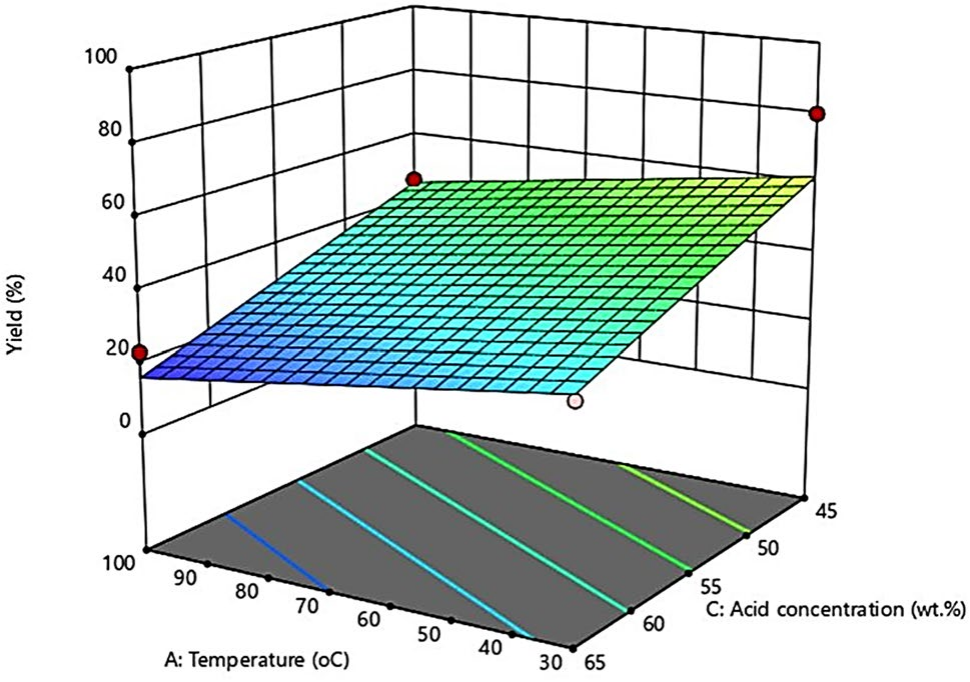
Interaction effect between hydrolysis temperature and concentration.

**Figure 9:**
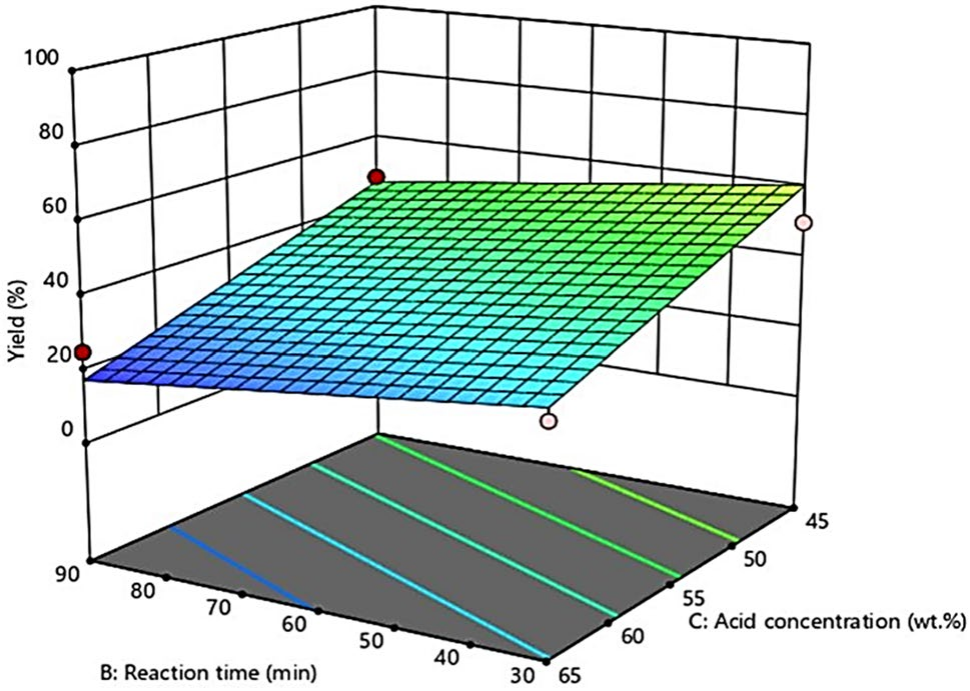
The interactional effect between reaction time and concentration of sulfuric acid.

Generally, the estimation of the combined influence of independent variables on the CNCs yield and the identification of various forms of the variable test interactions are usually done by the response surface plot. Figures 7, 8 and 9 depict the three-dimensional (3D) form of the response surface model, evidently showing the interactive effects of the three factors on the CNCs yield.

#### Interactive effect between hydrolysis temperature and hydrolysis time

Figure 7 shows the 3-D surface plot of the influence of interaction between temperature and hydrolysis time on the yield of CNCs, at constant sulfuric acid concentration of 55 wt%. The developed model revealed negative effects of the hydrolysis time and hydrolysis temperature on the yield of CNCs. It could be observed that, an increase in the hydrolysis temperature from 30 to 100 °C, decreased the yield of CNCs progressively. Similarly, about 70% CNCs yield was obtained during the initial 30 min of the acid hydrolysis, at the minimum hydrolysis temperature of 30 °C. However, when the hydrolysis time was prolonged to 90 min, the yield declined to 18%. This could be because there was insufficient energy in the form of heat to overcome the cellulose macromolecules' strong bonding system and main force, enabling the cellulose structure to favour its unchanging crystalline form. Thus, the hydrolysis process was not likely to be favoured by increasing the hydrolysis time. A study conducted by Xiang et al.^40^ corroborates these findings. When corncob-derived cellulose was treated at 100 °C for 30 min, the nanocellulose yield was considerably reduced from 70 to 31%. At the initial stage, there was an assumption that the cellulose crystalline structure has collapsed, and there was transformation of the majority of the solid cellulose into liquid reducing sugars or further broken down into acid by-products^41^.

#### Interactional effect between hydrolysis temperature and concentration of sulfuric acid

The interaction effect between the hydrolysis temperature and the acid concentration at a constant hydrolysis time of 60 min on the CNC yield is presented in Fig. 8. It can be observed that the CNCs yield decreased with an increase in hydrolysis temperature, an increase in hydrolysis time, and an increase in sulfuric acid concentration. This result is consistent and comparable to the previous findings of Hamid et al.^42^. This observation could result from the hydrolyzing catalyst breaking of the cellulose amorphous domains, reducing the CNCs yield. The lowest product yield (18%) was obtained when the hydrolysis was performed at high hydrolysis time and low hydrolysis temperature (30 °C, 90 min, 55 wt. %), resulting in extreme decomposition of cellulose and unwanted side reactions such as dehydration^43^.

#### Interaction effect between reaction time and acid concentration

Figure 9 represents the CNCs yield as a function of hydrolysis time and acid concentration, at a constant hydrolysis temperature of 65 °C. Increasing the concentration of acid and the hydrolysis time to 65% and 90 min, respectively, decreased the CNCs yield from 48 to 32%. The hydrolyzing catalyst penetrated the cellulose matrix and enabled the initiation of the hydrolysis process owing to the increased hydrolysis time. Therefore, increased reaction time can result in the breakdown of the cellulose, reducing the surface area of the hydrolyzed product and, consequently, reducing the effectiveness of the acid hydrolysis^44^. Obviously, treating the corncob-derived cellulose with sulfuric acid stimulates the hydrolytic degradation of cellulose, thereby resulting in the gradual reduction in the CNCs yield, as depicted in Fig. 9. Furthermore, this may be because of the over degradation of cellulose into an undesired product at increased acid concentration and an elongated hydrolysis time. The result is consistent with the literature^39,44^.

### Optimization of the process conditions

**Figure 10:**
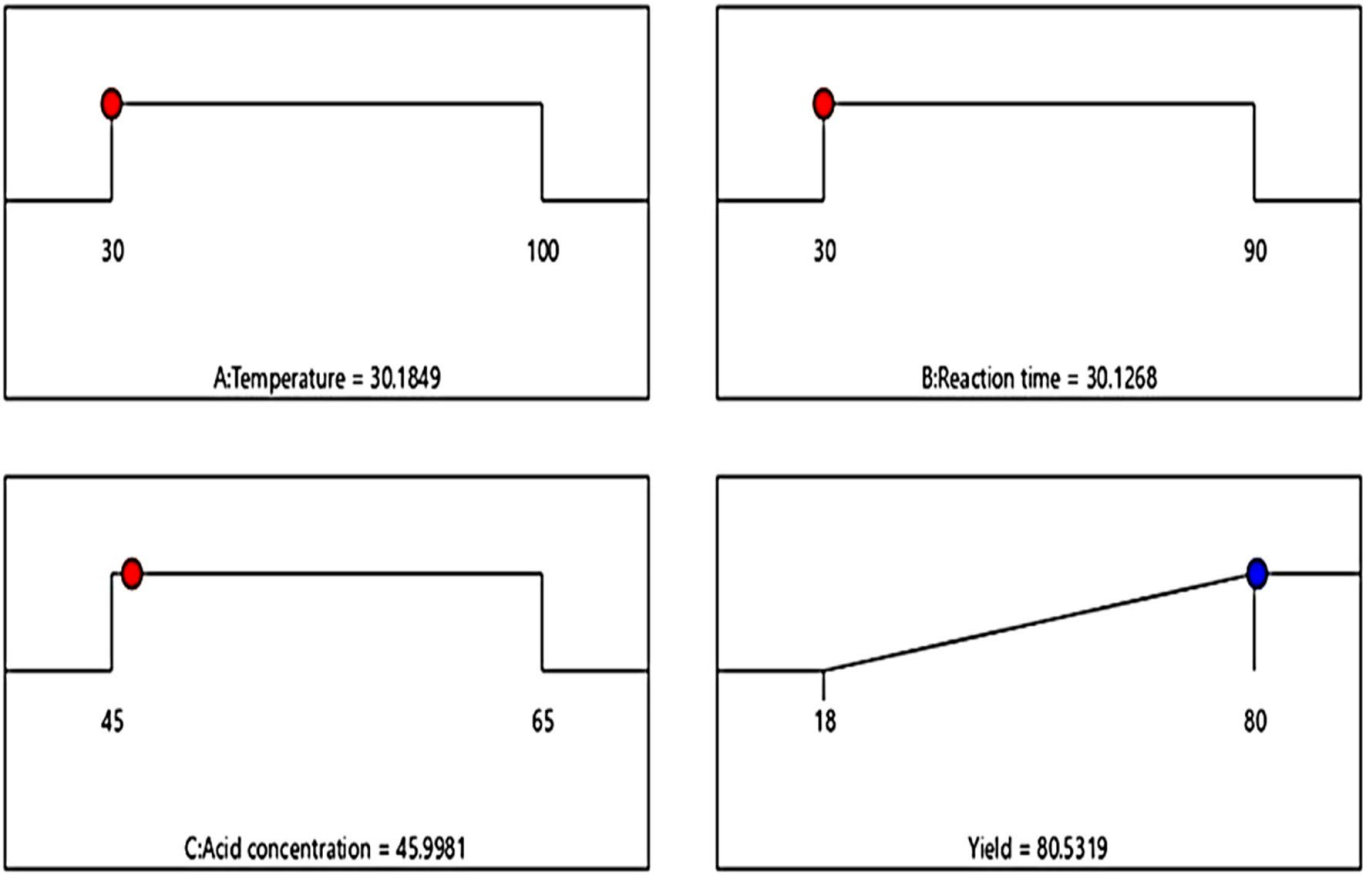
Optimized process condition.

The principle of optimization helps us to take full advantage of the economic benefit from reducing the cost of production. The response variable (CNCs yield) was set at optimum, while the process parameters were set at their minimum values^45^. Figure 10 shows the optimized process conditions. A multiple response method also known as desirability (D) function was used to carry out the parametric optimization. In line with the experiments performed in Table 5, the optimum yield of CNCs of 41.8% was achieved at the hydrolysis temperature, time, and acid concentration of 30.18 °C, 30.13 min, and 45.98 wt%, respectively. A predicted CNCs yield of 80.53% was obtained under the interaction conditions. Desirability of 1 was attained, indicating the achievement of the aim of optimizing and enhancing the CNCs yield.

**Table 5:** Result of experimental check of the predicted yield at optimal conditions.

Optimized conditions	Temp (°C)	Time (min)	Conc. (wt%)	Yield (%)
Predicted	30.18	30.13	45.998	80.53 ± *0.35*
Experimental	30.18	30.13	45.998	79.73 ± 0.17

### Experimental check of predicted yield at the optimal conditions

An experiment was conducted at maximum hydrolysis conditions of acid concentration (45.998 wt%), reaction time (30.13 min), and reaction temperature (30.18 °C) obtained from the parametric optimisation to check the obtained optimum yield. The experiments were therefore performed in triplicate at the above specified optimum process conditions predicted by the model to ensure accuracy of results, reduce experimental errors, and validate the maximum conditions predicted by the response surface methodology model results. As it can be seen from Table 5, the mean yield percentage of 79.73 ± 0.17% was obtained, under the predicted conditions. A desirability function was then employed to correlate the data obtained from the optimization analysis with the CNCs yield obtained from the experiment. The modelled results were within 95% confidence interval, which is consistent with the experimental result, hence confirming the appropriateness and correctness of the model.

### Comparison of the optimum yield of CNCs in this study with literature

Table 6 compares the results obtained in this study with relevant literature on the optimized parameters for obtaining the maximum yield of CNC from waste biomass. Wijaya et al.^12^ investigated the optimization of CNCs yield from bamboo shoots by CCD, using Response surface methodology. A maximum CNC yield of 50.61% at 39 °C reaction temperature and 54.73 wt% acid concentration was obtained. Wijaya and co-authors' results are compared with the results obtained in this recent study, where a maximum CNCs yield of 80.53% was attained at a reaction temperature of 30.18 °C, the reaction time of 30.13 min, and acid concentration of 45.998 wt%. It can be observed that the CNCs yield is about 37.7% more than Wijaya et al.^12^ even though the experimental conditions (reaction temperature and acid concentration) were higher, which could make the CNCs isolation process energy-intensive and costly. In addition, BBD was used in this current study with a minimum of 17 experiments, while 20 designed experiments were obtained by using CCD in Wijata et al.^12^, which takes more time to complete than DOE by BBD. More so, BBD contains fewer design points than CCD, making them less expensive to operate, and BBD ensures that no one factor is set to its maximum value at the same time.

**Table 6:** Comparison of results with literature.

Waste biomass	Temp (°C)	Time (min)	Conc. (wt%)	Yield (%)	DOE	Refs
Corncobs	45.00	59.93	61.66	41.74	CCD)	Demewoz^15^
Bamboo shoots	39	–	54.73	50.61	CCD)	Wijaya et al.^12^
Kraft pulp (Softwood)	58	60	60	60.00	CCD)	Kandhola^45^
Rice straw	30	300	75	90.28	CCD)	Thakur et al.^13^
Brewer's spent grain	51.01	41.07	50.57	43.24	CCD)	Matebie et al.^39^
Corncob	30.18	30.13	45.998	80.53	BBD)	This study

Likewise, Thakur et al.^13^ studied the optimization of synthesis of CNC from rice straw via sulfuric acid hydrolysis by RSM using CCD for the design of the experiment. An improved yield of CNCs (90.28%) was obtained at optimum conditions of 30 °C, 75 wt% acid concentration and 5 h. The CNCs yield obtained by Thakur et al.^13^ is 10.9% more than what was obtained in this current study, although a high sulfuric acid concentration was used, which could contribute to environmental pollution and the longer hydrolysis time (5 h) used could make the process costlier than this current study. About 40.94% CNCs yield was achieved in a study by Demewoz^15^ to investigate the extraction, characterization, and optimization of CNCs from corncobs using response surface methodology through CCD. The optimal conditions were 61.66 wt. % acid concentrations, reaction temperature 45 °C, and 59.92 min hydrolysis time. The CNCs yield in this current study is 49% higher than what was obtained by Demewoz^15^. These observations informed the operating conditions used in this study improved the yield of CNCs.

Therefore, the results documented in this study are comparable to relevant literature and have shown an improved CNC yield of 80.53% at reduced experimental conditions using the Box-Behnken to design the experiment, compared to previous studies where CCD was utilized.

## Conclusions

In this study, CNCs were successfully isolated from waste South African corncob via pretreatment technique and then acid hydrolysis. The SEM images showed that acid hydrolysis changed the surface morphology of the raw corncobs. The presence of pores can be observed on the surface of the acid-hydrolyzed cellulose fibers when compared to the raw corncob. FTIR spectra confirmed the functional groups on the surface of the raw corncobs and the isolated CNCs. The XRD spectra showed an improved crystallinity of the CNC after acid hydrolysis of the corncob-derived cellulose from 57.67% to 79.11%. BBD reduced the number of experiments compared to CCD explored in the literature, thereby saving time, and resources.

To understand the effect of hydrolysis conditions on the CNC yield, the BBD was utilized to design the experiment. Using the regression model developed from the data generated, the RSM approach was used to study the effect of the considered variables on the yield of CNCs. Lastly, the desirability function was employed to study the parametric optimization of the hydrolysis using the validated regression model as the objective function. The obtained results reveal that the CNCs yield increased with decreasing acid concentration and reduced reaction temperature and reaction time. Results reveal that the optimal hydrolysis conditions, within the limit of the experimental error and the constraints, were: acid concentration of ~ 46 wt% acid concentration, hydrolysis time of 30.13 min, and hydrolysis temperature of 30.18 °C. At these optimal conditions, the optimum predicted CNC yield and the optimum experimental CNC yield were 80.53 ± 0.35% and 79.73 ± 0.17%, respectively.

The results documented in this study demonstrated that corncob could be used as a raw material to produce cellulose nanocrystal that could potentially be utilized in numerous applications such as pharmaceuticals, membranes technology for environmental remediation, nanocomposite synthesis, etc. Therefore, this study shows that the isolation of cellulose nanocrystal from waste corncob via acid hydrolysis could be efficiently developed on an industrial scale. Finally, the application of waste corncob in the isolation of CNCs could help in the attainment of zero waste generation goals, also known as circular economy, minimizing waste management problems and environmental challenges in the process.

## Data Availability

All data underlying the results are available as part of the article and no additional source data are required.
